# Assessing the Acetabular Cup Implant Primary Stability by Impact Analyses: A Cadaveric Study

**DOI:** 10.1371/journal.pone.0166778

**Published:** 2016-11-28

**Authors:** Adrien Michel, Romain Bosc, Jean-Paul Meningaud, Philippe Hernigou, Guillaume Haiat

**Affiliations:** 1 CNRS, Laboratoire de Modélisation et de Simulation Multi-Echelle, Créteil, France; 2 INSERM U955, IMRB Université Paris-Est, Créteil, France; 3 Service de Chirurgie Orthopédique et Traumatologique, Hôpital Henri Mondor AP-HP, CHU Paris 12, Université Paris-Est, Créteil, France; Harvard Medical School/BIDMC, UNITED STATES

## Abstract

**Background:**

The primary stability of the acetabular cup (AC) implant is an important determinant for the long term success of cementless hip surgery. However, it remains difficult to assess the AC implant stability due to the complex nature of the bone-implant interface. A compromise should be found when inserting the AC implant in order to obtain a sufficient implant stability without risking bone fracture. The aim of this study is to evaluate the potential of impact signals analyses to assess the primary stability of AC implants inserted in cadaveric specimens.

**Methods:**

AC implants with various sizes were inserted in 12 cadaveric hips following the same protocol as the one employed in the clinic, leading to 86 different configurations. A hammer instrumented with a piezoelectric force sensor was then used to measure the variation of the force as a function of time produced during the impact between the hammer and the ancillary. Then, an indicator *I* was determined for each impact based on the impact momentum. For each configuration, twelve impacts were realized with the hammer, the value of the maximum amplitude being comprised between 2500 and 4500 N, which allows to determine an averaged value *I*_*M*_ of the indicator for each configuration. The pull-out force *F* was measured using a tangential pull-out biomechanical test.

**Results:**

A significant correlation (R^2^ = 0.69) was found between *I*_*M*_ and *F* when pooling all data, which indicates that information related to the AC implant biomechanical stability can be retrieved from the analysis of impact signals obtained in cadavers.

**Conclusion:**

These results open new paths in the development of a medical device that could be used in the future in the operative room to help orthopedic surgeons adapt the surgical protocol in a patient specific manner.

## Introduction

Press-fit procedures are more and more often employed in hip surgery and aim at inserting cementless implants in slightly undersized bone cavities [1, 2]. However, aseptic loosening represents one of the main causes of failure of cementless orthopedic implants [3, 4]. Micromotions at the bone-implant interface may induce the development of fibrous tissue around the implant, which could be responsible for aseptic loosening [5–8]. The implant primary stability is a major determinant of the surgical success of hip arthroplasty surgery [9]. However, a compromise has to be found for the implant stability and in particular regarding the pre-stressed state of bone tissue. The level of stresses due to the press-fit insertion should be sufficiently important in order to avoid excessive relative micromotions of bone tissue compared to the implant surface. However, the amplitude of the stress field should not exceed a certain threshold in order to avoid bone necrosis which may be caused by a too important magnitude of the stress field in the acetabulum [10]. Moreover, a compromise has to be found for the number and the magnitude of the impacts produced by the surgeon with the hammer on the ancillary during the implant insertion, which must be “sufficient” to allow adapted implant stability, but not “too high” to avoid acetabular bone fracture [11].

Despite the importance of assessing the acetabular cup (AC) implant primary stability, there is a lack of adapted tool to assess this property in the operative room. Medical imaging techniques such as microcomputed tomography or magnetic resonance imaging cannot provide quantitative information on the bone-implant interface because of diffraction phenomena due to presence of metals [12, 13]. Currently, surgeons evaluate the implant stability empirically by listening to the sound produced when impacting the ancillary with the orthopedic hammer [14].

While biomechanical tests have been used to assess the AC implant primary stability [10, 15, 16], such approaches remain destructive and are restricted to *in vitro* or animal studies. Cristofolini et al. (2006) have developed a device that can measure micromotions at the bone implant interface while applying a 20 N.m torque to the implant. Despite encouraging results, the set-up has encountered reproducibility issues [17]. Vibrational techniques have been used to estimate the implant primary stability [18, 19]. However, such approach remains difficult to be used in the operative room and to the best of our knowledge, no tool is yet available to help orthopedic surgeons assessing the implant primary stability intraoperatively.

Recently, our group has shown that the analysis of the variation of the force as a function of time produced during the impact between the hammer and the ancillary can be used in order to retrieve the implant insertion properties. By realizing reproducible mass drops, the contact duration was found to be a useful indicator to follow the AC implant insertion [20]. A second indicator based on the impact momentum was then shown to be more accurate [21] to follow the AC implant insertion conditions and to assess the AC implant primary stability [22]. In order to develop a device that could be used intraoperatively in a patient specific manner, a hammer was then instrumented and the technique was adapted to predict the AC implant stability using such impact hammer [22, 23]. A numerical model has also been used to understand the phenomena occurring during the impaction and the numerical and experimental results were compared [24]. However, the impact hammer has not been tested in a configuration close to that of the operating room, which is of interest for the future development of such medical device.

The aim of this study is to evaluate whether the analysis of impacts signals corresponding to the variation of the force as a function of time obtained during impacts between a hammer and the ancillary could be employed in conditions closer to these of the operating room, which would allow future *in vivo* measurements. To do so, the experiments were carried out with cadaveric specimens because such situation allows the pull-out force to be measured, which is not the case when dealing with patients. Twelve human hips were tested *ex vivo* in different configurations detailed below.

## Material and Methods

### 1. Cadaveric hip specimens and acetabular cup implant

Six cadavers were used in this study, which was approved by the ethical committee of the Surgery School of the Fer à Moulin (Paris, France), which is a French institution who handles cadavers for research and education purposes. None of the authors had access to patient data identifications. The Ethics committee did not ask for any consent of the next kin in the present case. Both hips of each cadaver were tested, resulting in a total of 12 hips studied herein. The surgery was then realized similarly as in clinical conditions. All cadavers were placed in lateral decubitus position. A posterior approach to the hip joint was performed in all cadaveric specimens. After opening and exposing the joint capsule, the femoral neck was osteotomized and the femoral head was removed.

Four different AC implants (Cerafit Uncemented Hip Prosthesis, Ceraver, Roissy, France) with different diameters (49, 51, 53 and 55 mm) were used as far as practical for each hip sample. All AC implants were screwed to an ancillary and inserted using successive impacts, as described in more details in section 2.5.

### 2. Instrumented hammer

**Fig 1:**
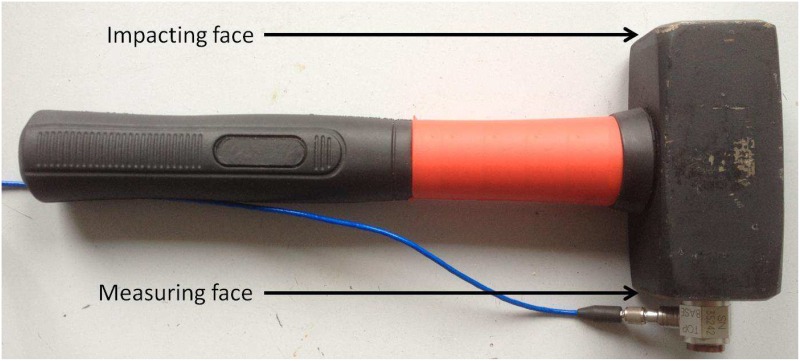
Hammer instrumented with a dynamic piezoelectric force sensor (208C05, PCB Piezotronics, Depew, New York, USA) screwed on the hammer impacting face.

**Fig 2:**
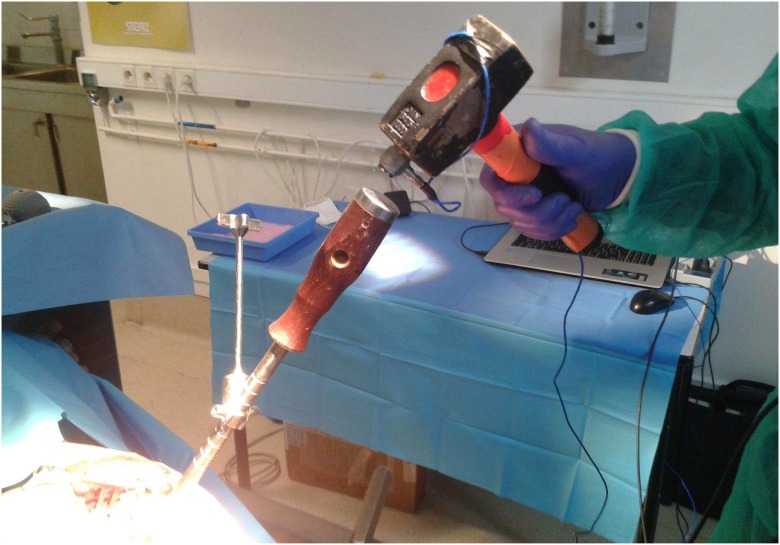
Illustration of the AC implant impact carried out with the measuring face of the impact hammer during the measurement protocol.

The two opposite faces of an instrumented hammer (*m* = 1.3 *kg*) were used in this study (see Fig 1), similarly as in [23]. The first face (referred to as impaction face) was used to insert the AC implant within bone tissue, as described in more details in subsection 2.5. The opposite side of the hammer (referred to as measuring face) was equipped with a dynamic piezoelectric force sensor (208C05, PCB Piezotronics, Depew, New York, USA) screwed on the hammer impacting face, similarly as what has been done in [23]. Fig 2 shows the measurement configuration considered when using the impact hammer.

When using the measuring face, the time variation of the force *s(t)* applied between the instrumented hammer and the ancillary was recorded using a data acquisition module (NI 9234, National Instruments, Austin, TX, USA) with a sampling frequency of 51.2 kHz and a resolution of 24 bits. A labview (National Instrument, Austin TX, USA) interface was used to record the signals corresponding to the force applied between the hammer and the ancillary for a duration of 2.5 ms.

### 3. Signal processing

The radiofrequency (*rf*) signals *s(t)* corresponding to the variation of the force as a function of time produced during the impact between the hammer and the ancillary was processed using a dedicated signal processing method described in [23]. A threshold of 30 N was used to identify the beginning of the impact. For each configuration, a quantitative indicator *I* referred to as impact momentum [22] was determined following:

$$I=\;\frac{1}{A_{0}\left(t_{2}\;-\;t_{1}\right)}\;\int_{t_{2}}^{t_{1}}s\left(t\right).dt,$$ (1)

where *A*_*0*_ *= 400 N*, *t*_*1*_ *= 0*.*35 ms and t*_*2*_ *= 1*.*11 ms*. The choice of the values *t*_*1*_ and *t*_*2*_ will be discussed in section 4. Matlab (The Mathworks, Natick, MA, USA) was used to analyze the data.

### 4. Tangential stability tests

**Fig 3:**
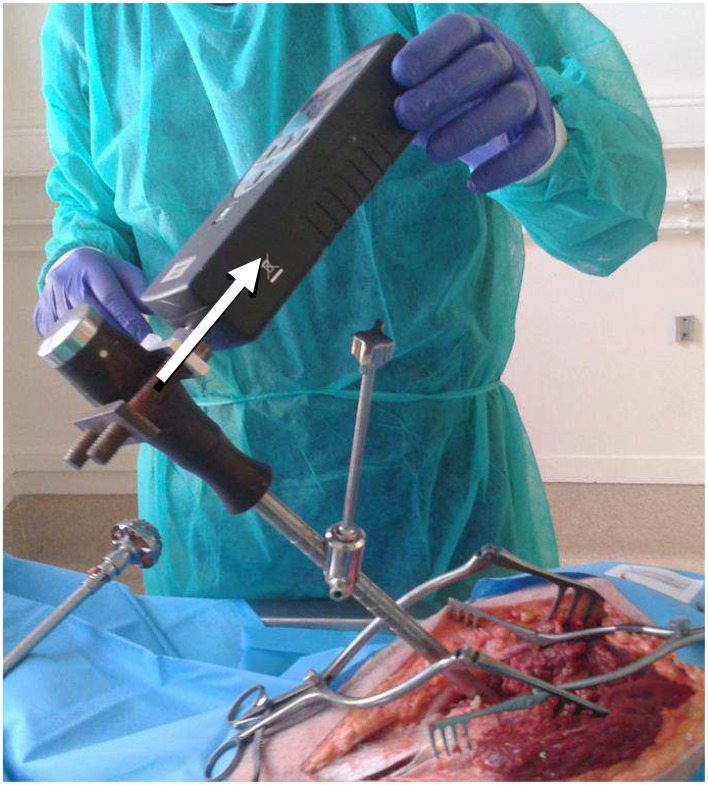
Illustration of the tangential stability testing configuration (the arrow represents the direction of the force applied. The force is applied along the anteroposterior direction.

Similarly as what was done in previous studies [1, 15, 22, 23], the AC implant primary stability was assessed using a tangential stability testing configuration shown in Fig 3. While the hip was manually held, a numerical dynamometer (DFX2-050-NIST, AMETEK, Elancourt, FRANCE) fixed at the top end of the ancillary at a constant location underwent a gradually increasing force (step of around 8 N.s^-1^) until the AC implant was extracted from the hip. The maximum value *F* of the force obtained during the removal of the implant from the acetabulum was then determined.

### 5. Experimental protocol

**Fig 4:**
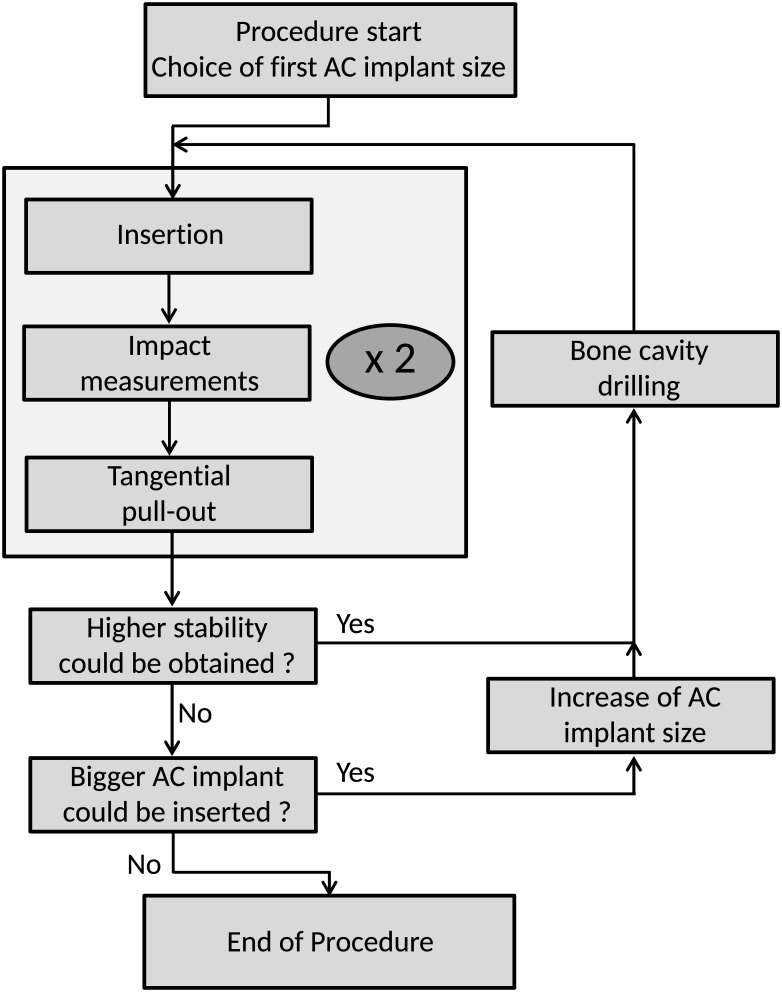
Schematic representation of the experimental protocol used for each hip of each cadaveric specimen.

The experimental protocol was carried out by an orthopedic surgeon for all cadaveric specimens, as described schematically in Fig 4. The protocol for this cadaveric study was submitted and approved by the scientific committee of the Ecole de chirurgie du Fer à Moulin. The protocol is based on empirical considerations determined by the surgeon in order to obtain a maximum number of configurations for each hip tested, which allows to optimize the use of cadaveric specimen, as required by ethical considerations. To do so, our approach consists in considering different AC implant sizes and to reproduce the experiments as far as practicable in each hip specimen, without damaging the acetabulum.

#### Initial implant insertion

The surgeon first chose the smallest AC implant size that could be inserted in each cadaveric hip, according to the acetabulum anatomy. A bone cavity was then reamed using the dedicated reamer provided by the manufacturer and imposing an interference fit equal to 1 mm. The AC implant was inserted into the acetabulum by impacting the ancillary with the impaction face of the hammer until the surgeon estimated that the implant could not be further inserted without damaging the surrounding acetabulum.

#### Measurement protocol

Once the AC implant has been inserted in the acetabulum, the average value of the indicator *I* was assessed using an “estimation protocol”, which is constituted by 3 successive impacts of the ancillary realized with the measuring face of the hammer. For each impact realized during the estimation protocol, the maximal amplitude of the force applied between the hammer and the ancillary was comprised between 2500 and 4500 N. The force signal *s(t)* was processed using Eq 1 in order to derive the value of the indicator *I* for each impact. The average value *I*_*m*_ of the 3 values of *I* obtained during the estimation protocol was then determined. This estimation protocol was then carried out three additional times to assess the reproducibility of the estimation of *I*_*m*_. Therefore, the estimation protocol was realized a total of four times, which corresponds to a total of 12 impacts realized with the measuring face of the hammer. Then, the average and standard deviation values (denoted *I*_*M*_ and *I*_*SD*_) obtained for the three values of *I*_*m*_ were determined. Note that *I*_*SD*_ corresponds to the reproducibility of the measurement carried out using the aforementioned estimation protocol. The choice of the total number of impacts (twelve) and of the number of estimation protocol realized will be discussed in section 4. The AC implant stability was then estimated with the tangential stability testing configuration described in subsection 2.4.

#### Repetition of the procedure

Once the AC implant was pulled out of bone tissue, it was inserted again into the same bone cavity, which did not undergo any additional reaming. Then, the measurement protocol described in the last paragraph was again carried out. Note that repeating the same procedure (including insertion, measurement and pull-out) with the same implant and without modifying the cavity does not necessarily lead to the same value of *I*_*M*_, *I*_*SD*_ and *F* because i) the implant may not be inserted similarly and ii) of bone abrasion in the surrounding bone tissue.

#### Possible modification of the cavity

At the end of these two procedures (leading to two values of *I*_*M*_, *I*_*SD*_ and *F*), the surgeon decided (based on empirical considerations) whether a higher stability could be obtained with the same implant. In this case, the surgeon drilled further with the same reamer if necessary and repeated the measurement procedure two times, as described above.

#### Possible change of the implant size

Once the surgeon estimated that a higher stability could not be obtained with the same AC implant, the surgeon decided if a bigger implant could be implanted in the same cavity. In this case, the same procedure described above was repeated with an implant having a diameter just slightly higher than the implant previously used.

This protocol resulted in a total of 86 different configurations (with different implants and cavity properties), corresponding to 86 values of *F*, *I*_*M*_ and *I*_*SD*_. The relationship between *I*_*M*_ and *F* when all data were pooled together was studied using a linear regression analysis.

## Results

Table 1 shows the number of experiments carried out for each hip considered following the protocol described in subsection 2.5, together with the diameter of the AC implant used. As far as we could notice, no fracture was experienced in any of the hip tested.

**Table 1:** Number of configurations considered for each cadaveric specimen and each hip. The AC implant diameters are also indicated. r and l denote right and left, respectively.

	Cadaver #					
AC diameter	1 (r/l)	2 (r/l)	3 (r/l)	4 (r/l)	5 (r/l)	6 (r/l)
49 mm	0/0	0/0	0/0	2/4	0/0	0/0
51 mm	2/2	2/0	0/0	2/4	2/2	2/0
53 mm	0/0	2/6	2/4	0/4	2/4	4/2
55 mm	0/0	2/4	4/8	2/4	2/2	2/2
Total	4	16	18	22	14	12

**Fig 5:**
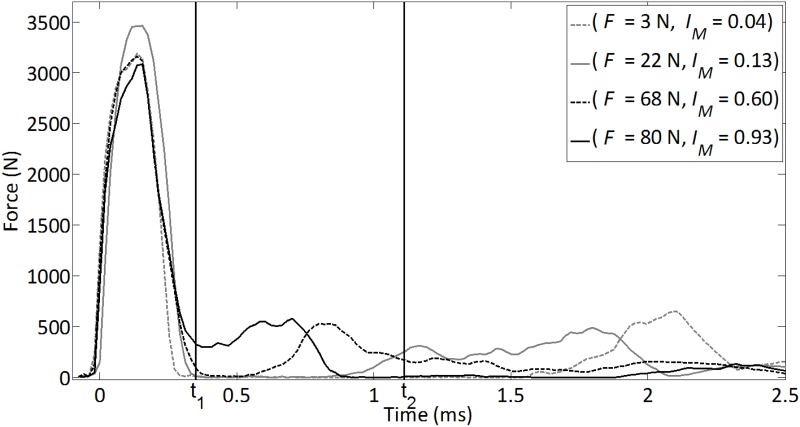
Four *rf* signals (corresponding to the time variation of the normalized averaged force signal) with the corresponding stability of the AC implant. The two lines in Fig 5 show the time window where the indicator is determined (see Eq 1).

The different behaviors of the rf signals obtained for various stability conditions of the AC implant are shown in Fig 5. The two vertical lines in Fig 5 show the time window used to determine the indicator (see Eq 1). The average values of all *rf* signals used to compute *I*_*M*_ are shown in Fig 5 for four different stability conditions. The corresponding values of the pull-out forces and of the indicator *I*_*M*_ are also indicated. Fig 5 shows that the *rf* signals obtained are significantly different, although all curves shown in Fig 5 exhibit a “secondary rebound”, corresponding to a second maximum occurring after the initial peak which has the highest energy. Such secondary maximum is shown to occur at a time which decreases when the implant stability increases (around 2.1 ms for an implant stability equal to 3 N and around 0.7 ms for an implant stability of 80 N). Note that these results are consistent with those obtained in [21–23].

The average value of the standard deviation *I*_*SD*_ was found to be equal to 0.05, while its maximum (respectively minimum) value was equal to 0.17 (resp. 0.002). These results provide an order of magnitude for the reproducibility of the measurement protocol consisting in three impacts.

**Fig 6:**
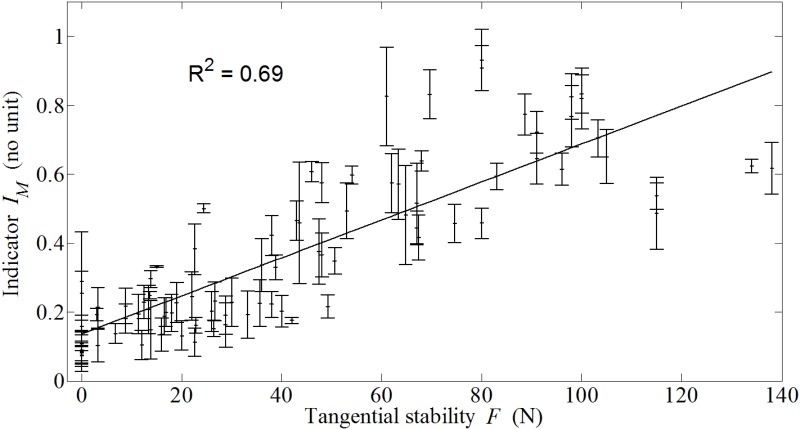
Variation of the mean value *I*_*M*_ of the indicator as a function of the pull-out force *F* obtained with the tangential stability test for all data pooled together. The error bars correspond to *I*_*SD*_, the standard deviation obtained for the indicator.

Fig 6 shows the results corresponding to the variation of *I*_*M*_ as a function of *F* obtained when all data obtained from all samples are pooled together. A linear regression analysis shows a significant correlation (R^2^ = 0.69) between the indicator *I*_*M*_ and the tangential stability *F*.

## Discussion

The originality of the present study is to use an instrumented hammer to evaluate the AC implant primary stability under conditions closed to the operating room. Previous studies have considered the use of vibrational techniques to assess orthopedic implant insertion conditions. The insertion endpoint [25] and primary stability [18, 19, 26] of the AC and hip stem implants could be assessed intraoperatively with a similar precision. However, to the best of our knowledge, it remains difficult to assess the AC implant primary stability during the surgery. The interference fit was chosen equal to 1 mm according to the manufacturer requirements and to the results found in previous studies [4, 27, 28]. Such value of the interference fit usually allows to provide an adequate stability conditions to the AC implant. Moreover, the primary stability was evaluated using a tangential pull-out test, which has the advantage of being adapted to the configuration of this cadaveric study [10, 15, 29]. The values of stability were obtained in the range [0–140 N] which is in agreement with the range found in the literature [1, 30, 31]. The implant stability also depends on the sinking relative to the edges of the cup because a cup in a *coxa profunda* is different from a cup implanted in hip dysplasia, where a part of the wall may be discovered [32]. However, the sinking relative to the edges of the cup was not investigated herein because i) this has already been done in a previous *in vitro* study [20] which showed that the indicator is sensitive to the cup insertion properties and ii) it is difficult to quantitatively determine the cup sitting in cadavers.

The same force was not used for all insertions of the AC implants. The force imposed to the ancillary through the hammer is applied through various impacts. The number and energy of impacts used to insert the AC implant depend not only on the type of bone (sclerotic, osteoporotic…), but also on the type of the AC implant and on the geometry of the cavity. The number and energy of impacts are currently determined empirically by the surgeons. The aim of the medical device under development is to provide a decision support system to the surgeon, which will allow to find a compromise between too important impact number and energy, which may lead to bone fracture and too low impact number and energy, which may lead to unstable implants. Therefore, the proposed methodology consists in inserting the AC implant following the procedure used in the clinic, so that the surgeon uses his proprioception to adapt the impact number and energy. Then, the impact hammer is tested and the pull-out force is measured. Therefore, the impact energy is not always the same for all AC implants. However, the aim of this paper is not to relate the impact energy and the indicator but to relate the pull-out force and the indicator. Note that in previous papers by our group [20–22], we used a device producing reproducible mass falls in order to control the impact energy and force. However, such device cannot be used in the clinic, which is precisely why we chose to follow the same protocol as the one used in the clinic in the present paper.

It would be interesting to study the variation of the stability as a function of the number of strokes. However, this point is out of scope of the present study since we aim at validating the use of the impact hammer. Therefore, we choose to test the performances of the impact hammer on AC implants inserted in an optimal manner according to the surgeon’s proprioception, similarly as what is done in the clinic. Moreover, we choose not to investigate the variation of the stability as a function of the number of strokes because it would imply to carry out many pull-out tests, which would lead to a reduction of the number of tested configurations indicated in Table 1, due to abrasion phenomena [21]. Note that similar analyses have been carried out *in vitro* in a previous study [20] where we evidenced a significant relationship between the indicator and the number of strokes applied to the AC implants.

Table 1 shows that the total number of experiments that were carried out for each cadaver and each hip was equal to 7.2 ±4. The results indicate that there is a strong heterogeneity of the number of test realized according to the hip and to the cadaver, which may be explained by inter-individual anatomical variability.

The results shown in Fig 5 indicate that after the initial maximum of the force as a function of time, secondary maxima are obtained between around 0.4 and 2.5 ms, with an approximate amplitude of around 15% of the amplitude of the first maximum. These results are qualitatively similar to the results obtained *in vitro* using a device allowing reproducible mass falls [20–22] as well as the same impact hammer [23]. However, the relative amplitude of the secondary maxima compared to that of the first maximum obtained herein (around 15%) is lower compared to the results obtained in [23] (around 40%). This difference in amplitude of the secondary maxima may be explained by the presence of soft tissues around the acetabulum and to the fact that the acetabulum is not clamped into a rigid mass, which decreases the rigidity of the system and induces an increase of damping effects that may both be responsible for higher absorption of resonance effects (which are associated to these secondary maxima) [21, 28]. However, more work is needed to understand and quantify the influence of the soft tissues on the impact signals recorded by the force sensor. Moreover, Fig 6 shows that the frequency of the signal comprised between 0.4 and 2.5 ms qualitatively increases when the pull-out increases, which is consistent with the analytical model developed in [21] and with the results obtained in [28]. As a result, the correlation coefficient between the pull-out force and the indicator obtained in the present study (r^2^ = 0.69) is qualitatively similar to the results obtained in vitro with the impact hammer (r^2^ = 0.83) [23] and with the device allowing reproducible mass falls (r^2^ = 0.65) [22].

Several parameters were chosen empirically. First, the impacts realized during the estimation protocol had a maximum value of the amplitude comprised between 2500 and 4500 N following the results obtained in [22], which corresponds to a compromise defined as follow. This interval (2500–4500 N) was chosen so that the impacts have a sufficiently low energy in order to avoid modifications of the implant insertion and/or of the bone-implant interface properties. This level of amplitude should be lower than typical forces used to insert the AC implant *in vivo* (comprised in the interval [6000 N; 15000 N] [33]). However, lower amplitudes were not considered because it did not allow to obtain information on the bone implant interface (data not shown), which might be due to insufficient stress levels which does not allow to assess the mechanical response of the bone-implant interface.

Second, we chose to consider three impacts in the measurement protocol, which was repeated four times (leading to a total number of twelve impacts), as a result from different compromises. Firstly, the choice of a number of impacts equal to three used in the measurement protocol was made to obtain a sufficiently high value to allow an averaging of possible errors due to difference of impact conditions and a sufficiently low value to minimize the complexity and the time necessary to carry out the future measurements in the operating room. Secondly, the total number of impacts realized for the stability assessment was set equal to twelve following previous results [23], which allows to reach a compromise between and a sufficiently high number to obtain enough impacts to be able to assess the reproducibility of the measurements and a sufficiently low number in order i) to decrease fracture risk in the acetabulum and ii) to avoid possible modifications of the bone-implant interface.

Third, the impact momentum was defined approximately in the same time interval as in previous *in vitro* studies [21, 22]. The values of *t*_*1*_ = 0.35 *ms* and *t*_*2*_ = 1.11 *ms* were adapted according to the differences between the different *rf* signals as the one shown in Fig 3. An optimization study was then run to find the values for of *t*_*1*_ and *t*_*2*_ that maximize the correlation coefficient between *I* and *F*. Changing the values of *t*_*1*_ between 0.31 and 0.39 *ms* or the value of *t*_*2*_ between 1.07 and 1.15 *ms* did not affect significantly the results (less than 5% difference for R^2^, data not shown).

The present study has several limitations. First, possible variations of soft tissues thicknesses surrounding the acetabulum were not controlled. Soft tissue may be responsible for an attenuation of the vibrations caused by the impaction and therefore may affect the impact signals. Second, different sizes of AC implants were used. The implant size may affect the stability as well as the relationship between the stability and the impact signals. A study using the same implant size should be carried out in the future, which was not possible in the present study because we aimed at optimizing the use of cadavers by increasing the number of testing configurations, due to the ethical considerations. However, despite varying soft tissues properties and implant sizes, a significant correlation was obtained between the average value of the indicator *I*_*M*_ and the implant stability, which shows the feasibility of our approach. Third, only one hammer was used in this study and the same experiments should be done with different hammer (size, weight, shape…). Fourth, bone properties of cadaveric samples may be different from these of patients and the same set of experiments should be carried out in patients in the future. However, the assessment of the implant stability would then not be possible and one would then have to rely on the surgeon proprioception. Fifth, we did not realize pre-implantation CT-scan because this procedure is not carried out systematically in the clinic and we aimed at reproducing the usual clinical procedure. However, it would be of interest to determine whether bone quality affects the value of the indicator, which will be determined in future studies. Note that beside bone quality, other features such as implant and cavity properties also affect the AC implant stability.

## Conclusion

The results obtained in Fig 6 indicate a clear relationship between the indicator, which can be retrieved from the analysis of the impact, and the tangential stability, which indicates that impact analysis can be used in order to determine the AC implant primary stability. The approach described herein could be used in the future in the operating room to help the surgeon adapt the surgical strategy in a patient specific manner. In particular, obtaining information on the implant stability in a noninvasive manner could help the surgeon determine whether additional impacts are needed to insert the AC within bone tissue but also whether cavity modifications are needed or whether it would be preferable to choose an implant with a different geometrical configuration (e.g. different diameter). One of the most interesting advantages of an approach using an impact hammer employed with impact of relatively low amplitude is that the device under development will be easy to handle, noninvasive and will not require additional time for surgery. Future measurements should be done in patients to verify the accuracy of the method.
